# Peripheral lymphocyte subset variation predicts prostate cancer carbon ion radiotherapy outcomes

**DOI:** 10.18632/oncotarget.8389

**Published:** 2016-05-26

**Authors:** Zhang-Ru Yang, Ning Zhao, Jin Meng, Ze-Liang Shi, Bing-Xin Li, Xian-Wei Wu, Ping Li, Qing Zhang, Xun-Bin Wei, Shen Fu

**Affiliations:** ^1^ State Key Laboratory of Oncogenes and Related Genes, Shanghai Cancer Institute, School of Biomedical Engineering, Shanghai Jiao Tong University, Shanghai, China; ^2^ Department of Radiation Oncology, Shanghai Sixth People's Hospital of Jiao Tong University, Shanghai, China; ^3^ Radiation Oncology Center, Fudan University Shanghai Cancer Center (FUSCC), Shanghai Proton and Heavy Ion Center (SPHIC), Shanghai, China

**Keywords:** carbon ion radiotherapy, prostate cancer, peripheral lymphocyte

## Abstract

The immune system plays a complementary role in the cytotoxic activity of radiotherapy. Here, we examined changes in immune cell subsets after heavy ion therapy for prostate cancer. The lymphocyte counts were compared with acute radiotherapy-related toxicity, defined according to the Common Terminology Criteria for Adverse Events, and short-term local efficacy, defined based on prostate-specific antigen concentrations. Confirmed prostate cancer patients who had not received previous radiotherapy were administered carbon ion radiotherapy (CIR) in daily fractions of 2.74 GyE with a total dose of 63-66 GyE. Lymphocyte subset counts were investigated before, during and after radiotherapy, and at a 1 month follow-up. Most notable among our findings, the CD4/CD8 ratio and CD19+ cell counts were consistently higher in patients with a complete response (CR) or partial response (PR) to CIR than in those classified in the stable disease (SD) group (P<0.05 for both). But CD3+ and CD8+ cell counts were lower in the CR and PR groups than in the SD group. These results indicate that variations in peripheral lymphocyte subpopulations are predictive of outcome after CIR for prostate cancer.

## INTRODUCTION

Prostate cancer is the second most common cancer in men worldwide, with 1.1 million new cases estimated to have occurred in 2012 [1]. In China, the incidence of prostate cancer ranks sixth among male malignancies with a mortality rate that has increased more than 10-fold in the past two decades [2].

Radiation therapy continues to play an increasingly important role in the treatment of prostate cancer [3–5]. In recent years, the clinical use of charged particle therapy, mainly carbon ions and protons, has gained significant interest worldwide. Carbon ion beams present a Bragg peak also seen with protons, and provide a better dose distribution to the target volume via specified beam adjustments, such as utilizing the spread out Bragg peak (SOBP) [6]. In addition, carbon ions have greater potential to cause serious DNA damage, because its higher relative biological effectiveness (RBE), particularly at the distal edge of the Bragg peak may offer greater tumor control, and its smaller lateral penumbra may release a more conformal dose laterally and reduce the normal tissue damage. More importantly, carbon ions, like neutron beams, have a high RBE which results from high linear energy transfer (LET), and their efficacy of cytocidal action is approximately threefold greater than those of photons and protons [7].

The clinical application of proton and helium ion beams was started at the Lawrence Berkeley National Laboratory in the early 1950s, and clinical testing with heavy ion beams was initiated in 1970s. The first clinical trial of CIR for prostate cancer was initiated at the National Institute of Radiological Sciences (NIRS) in 1994. Subsequently, the most relevant trial of CIR for prostate cancer was performed, with a shorter duration of treatment than that conventionally used with photon radiation. The effectiveness and feasibility of CIR are well established [8]. Similar phase I/II trials have shown favorable results, supporting the clinical use of CIR [9].

Lymphocytes, one of the most radiation sensitive cell populations, account for approximately 30% of the normal human white blood cell population and are essential effector cells in anti-tumor immunity [10]. Changes in lymphocyte counts strongly correlated with carcinogenesis, tumor progression, and prognosis. Recent research demonstrates that photon radiotherapy induces severe treatment-related lymphopenia in a range of cancers [11-14], and this radiation-related lymphopenia is associated with early tumor progression and survival [15-17]. Numerous studies report the correlation between immunity and prognosis for cancers such as melanoma [18], ovarian [19], breast [20], lung [21, 22], esophageal [23], and prostate cancer [24]. These findings strongly suggest the importance of anti-tumor immunity in the prognosis of cancer. However, relatively few studies have examined the relationship between the immune reaction and CIR for prostate cancer patients.

In this study, we report the effect of CIR on peripheral blood lymphocyte subsets in Chinese prostate cancer patients. We quantified peripheral lymphocyte subsets in prostate cancer patients who had accepted CIR and analyzed the association between lymphocyte subset variations and both radiotherapy outcomes and treatment-related acute adverse effects including hematologic and urinary toxicities.

## RESULTS

### Patient and radiotherapy characteristics

Nineteen patients were enrolled (Table 1) from June to December 2014. CIR resulted in one of two outcomes: effective response (CR + PR) or ineffective response (SD). All 19 patients completed radiotherapy with CIR and were followed for at least 6 months. During the short-term follow-up, the local efficacy of radiation in the prostate was assessed by physical examination, MRI, TRUS, bone scintigraphy, ^11^C-CHO-PET CT, and PSA level. Five patients (27.78%) showed CR and 9 showed PR (50%), constituting 14 in total (77.78%) with an overall effective response (CR + PR), while 4 showed an overall ineffective response (SD) (22.22%). One patient's radiotherapy evaluation information was not acquired for an undisclosed reason. As presented in Table 1, the characteristics of the enrolled patients (including age, sex, and disease stage) were not associated with the local short-term efficacy of CIR for prostate cancer. Acute hematologic toxicity (leukopenia, anemia, and thrombocytopenia) occurred in 8 of the 18 cases (44.44%). Six of these 8 toxicities (75%) were degree I, and 2 (25%) were degrees II-III. Acute urinary adverse effects (increases in urinary frequency, noninfectious cystitis, and creatinine levels) occurred in 9 of the 18 cases (50%) and were graded as degree I. Acute organ adverse effects (excluding the urinary system) of the bone, soft tissue, skin, subcutaneous tissue, joints, and gastrointestinal system occurred in 8 of the 18 cases (44.44%). Six of these 8 cases (75%) were degree I, and 2 (25%) were degrees II-III. The short-term local efficacy of CIR was not associated with the rate of hematologic toxicities (P=0.6).

**Table 1:** Patient and treatment characteristics related to CIR for prostate cancer

Study population N=18	CR + PR N=14	SD N=4	P value
Age (years)			
Median	74	66	0.15
Range	62-80	61-75	
Risk stage			
I	1	1	0.45
II	7	1	
III	6	2	
Acute hematologic toxicity			
0	8	2	0.60
I	5	1	
II	1	1	
Acute urinary adverse effects			
0	6	3	0.26
I	8	1	
Acute organ adverse effects (not including the urinary system)			
0	7	3	0.60
I	5	1	
II–III	2	0	

CR + PR: patients with complete or partial response; SD + PD: patients with stable disease. PS: One patient's efficacy evaluation information was incomplete.

### Patient variation in all lymphocyte subset counts during CIR

**Figure 1:**
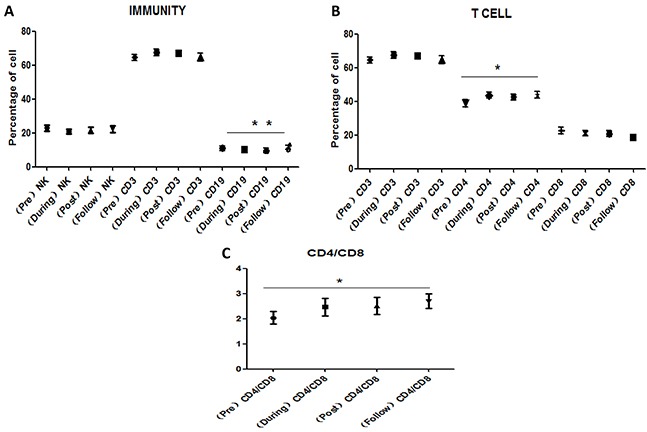
Variations in peripheral lymphocyte subpopulations for 4 time points **A.** Variations in NK, CD3+, CD19+ cells for 4 time points. CD19+ cells gradually decreased during radiotherapy but increased thereafter. **B.** Variations in CD3+, CD4+, CD8+cells for 4 time points. CD4+ cells increased during radiotherapy and follow-up. **C.** Variations in CD4/CD8 ratio for 4 time points. CD4/CD8 ratio increased during radiotherapy and follow-up. Data are presents as mean value of lymphocyte subset counts with the standard error of the mean (SEM). [Pre: pre-radiotherapy, During: during radiotherapy (after 10 fractions of CIR), Post: post-radiotherapy (the day when the full course of radiotherapy was completed), and Follow: follow-up (1 month after the final fraction of CIR); post-treatment.]

Figure 1 presents the variations in lymphocyte subset (NK, CD3+, CD4+, CD8+, CD4+/CD8+, CD19+) cell counts for each patient at the following time points: pre-radiotherapy, during radiotherapy (after 10 fractions of CIR), post-radiotherapy (the day when the full course of radiotherapy was completed), and at follow-up (1 month after the final fraction of CIR). For future studies, we suggest that the ratio of each lymphocyte subset at any time point and pre-radiotherapy be reported as a measure of all lymphocyte subset (NK, CD3+, CD4+, CD8+, CD4+/CD8+, CD19+) variation for the patient population. CD19+ cells gradually decreased during radiotherapy (P<0.01) (Figure 1A) but increased thereafter (P<0.01). CD4+ cells (Figure 1B) and the CD4/CD8 ratio (Figure 1C) increased during radiotherapy and follow-up (P<0.05 for both), and CD4+ cells decreased slightly after the last fraction of radiation.

### Variations in all lymphocyte subsets correlated with short-term CIR efficacy

**Figure 2:**
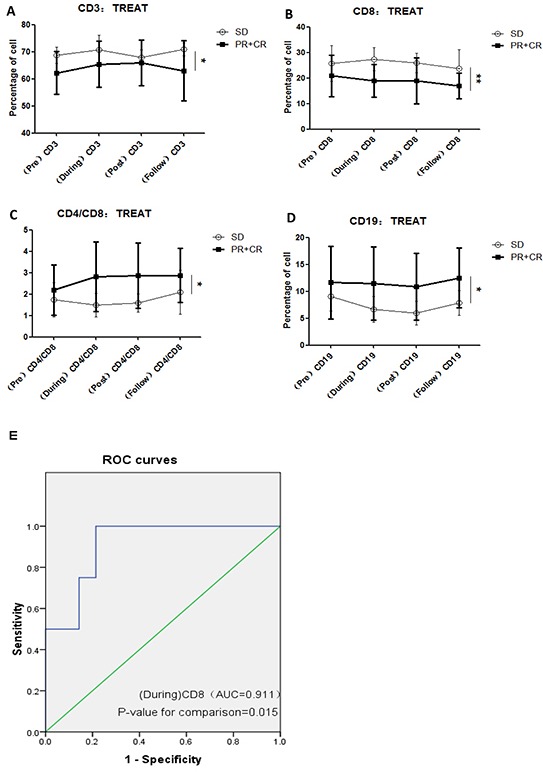
Association between variations in lymphocyte subsets and short-term efficacy of CIR **A.** CD3+ comparisons between effective response (CR + PR) and ineffective response (SD) groups. **B.** CD8+ comparisons between effective response (CR + PR) and ineffective response (SD) groups. **C.** CD4/CD8 comparisons between effective response (CR + PR) and ineffective response (SD) groups. **D.** CD19+ comparisons between effective response (CR + PR) and ineffective response (SD) groups. The CD4/CD8 ratio and CD19+ count in the CR + PR group were both higher than the corresponding concentrations in the SD group at the same points during radiotherapy and follow-up, while CD3+ and CD8+ cells in the CR + PR group were both lower than those in the SD group. **E.** (During) CD8+ cell analysis for the prediction of short-term efficacy of CIR.

A number of recent studies suggest that the immune system produces a synergistic therapeutic effect after radiotherapy [25]. Kobayashl et al. reported that the peripheral CD4/CD8 ratio was partially augmented by lycopene, a carotenoid, resulting in significant suppression of the development of spontaneous mammary tumors in mice [26]. Expression of CD3+ and CD4+ correlates with overall survival [27]. CD8+ cells exert anti-tumor immune effects via antigen-specific and antigen-nonspecific mechanisms [28]. Variation in number of CD4+ cells, the CD4/CD8 ratio, and number of CD19+ cells after CIR was observed in all 19 prostate cancer patients (Figure 2). In the effective response (CR + PR) group, CD3+ lymphocyte subpopulations in the peripheral blood gradually increased with the fractions of CIR administered, reaching a maximum after the final fraction of radiation (approximately 5-6 weeks) and decreasing thereafter. In the ineffective response (SD) group, the CD3+ cell levels first increased, then decreased to a minimum after the final fraction, and rebounded thereafter, while the CD8+ cells increased after 10 fractions (approximately 2-3 weeks) and then steadily decreased from radiotherapy to follow-up. In the (CR + PR) group, the CD4/CD8 ratio increased rapidly after 10 fractions of radiation and then increased slightly; however, in the SD group, the CD4/CD8 ratio decreased after 10 fractions, rebounded slightly following radiation, and then gradually increased after radiotherapy was complete. The CD19+ cells gradually decreased with increasing fractions of radiation, reaching a minimum after the final fraction of radiation, and then rebounded thereafter in both the (CR + PR) group and SD group.

As shown in Figure 2, the CD4/CD8 ratio and CD19+ cell counts in the CR + PR group were both higher than in the SD group during radiotherapy and follow-up (P<0.05 for both), while the CD3+ and CD8+ counts in the CR + PR group were lower than those in the SD group (P<0.05 and P<0.01, respectively).

Variations in lymphocyte subsets, such as CD3+, CD8+, and CD19+ cells and the CD4/CD8 ratio, between the two groups were considered potential predictive factors and were entered into logistic regression analysis. Appropriate cut-off levels were selected for their clinical significance. In a univariate analysis, short-term efficacy of CIR was associated with variations in CD3+ cells (P=0.0245), CD8+ cells (P=0.0012), the CD4/CD8 ratio (P=0.0122), and CD19+ cells (P=0.0130).

Multivariate analysis showed that during radiotherapy, the CD8+ cell count was an independent predictor of the short-term efficacy of CIR. The predictive value of CD8+ level on short-term efficacy was evaluated according to the area under the receiver operating characteristic curve (AUROC) (Figure 2). The ROC results (Table 2) indicate that the during-radiotherapy CD8+ cell count is relatively stable prognostic indicator for the short-term efficacy of CIR.

**Table 2:** Lymphocyte subsets for the prediction of short-term efficacy of CIR

AUROC: Area under the curve					
Test result variable	Area	Std. Error^a^	Asymptotic Sig.^b^	Asymptotic 95% confidence interval	
				Lower limit	Upper limit
(Pre) CD4/CD8	0.357	0.167	0.396	0.030	0.684
(During) CD4/CD8	0.161	0.118	0.044	0.000	1.000
(Post) CD4/CD8	0.179	0.104	0.056	0.000	0.436
(Follow) CD4/CD8	0.321	0.166	0.288	0.000	0.649
(Pre) CD19	0.446	0.136	0.750	0.180	0.713
(During) CD19	0.295	0.125	0.222	0.051	0.539
(Post) CD19	0.277	0.122	0.184	0.038	0.515
(Follow) CD19	0.214	0.107	0.089	0.005	0.424
(Pre) CD3	0.714	0.122	0.203	0.475	0.953
(During) CD3	0.705	0.124	0.222	0.463	0.948
(Post) CD3	0.589	0.130	0.595	0.335	0.843
(Follow) CD3	0.696	0.119	0.243	0.463	0.930
(Pre) CD8	0.696	0.132	0.243	0.437	0.956
**(During) CD8**	**0.911**	**0.072**	**0.015**	**0.000**	**1.000**
(Post) CD8	0.875	0.083	0.026	0.000	1.000
(Follow) CD8	0.750	0.146	0.137	0.388	1.000

a In the non-parametric assumption

b null hypothesis: the real area = 0.5

### Variations in all lymphocyte subsets correlated with acute CIR-induced toxicity

**Figure 3:**
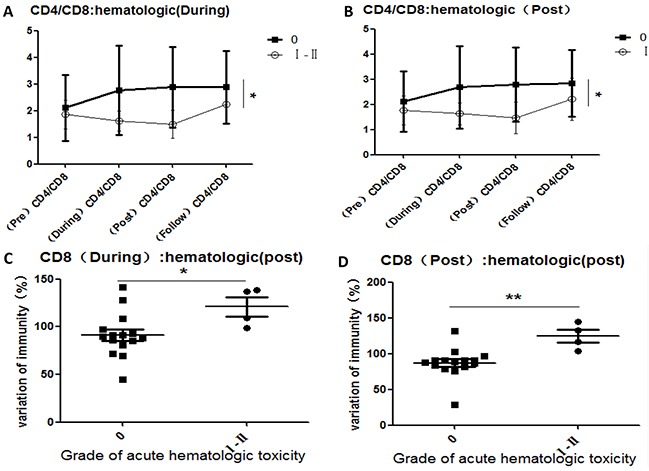
Correlations between variations in lymphocyte subsets and acute hematologic toxicity induced by CIR **A.** CD4/CD8 comparisons for the groups with grade 0 and grade I-II acute hematologic toxicity during 23/24 radiation fractions; **B.** CD4/CD8 comparisons for the groups with grade 0 and grade I-II acute hematologic toxicity after completion of radiotherapy; **C.** After 10 fractions of radiation, the ratio of CD8+ cell comparisons for the groups with grade 0 and grade I-II acute hematologic toxicity after completion of radiotherapy, assuming values of 100 at pre-radiotherapy; **D.** After completion of radiation, the ratio of CD8+ comparisons for the groups with grade 0 and grade I-II acute hematologic toxicity, assuming the values at pre-radiotherapy as 100. [Hematologic (During): acute hematologic toxicity occurring during 23/24 fractions of radiation; hematologic (Post): acute hematologic toxicity occurring after completion of radiotherapy; CD8 (During): the ratio of CD8+ cells after completion of the final radiation vs. that at pre-radiotherapy; CD8 (Post): the ratio of CD8+ cells after completion of the final radiation vs. that at pre-radiotherapy.]

**Figure 4:**
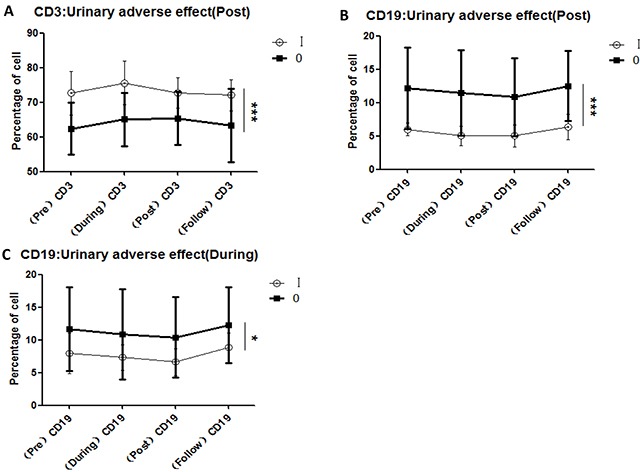
Correlations between variations in lymphocyte subsets and acute urinary adverse effects induced by CIR **A.** CD3+ cell comparisons for the groups with grade 0 and grade I acute urinary adverse effects after completion of radiotherapy; **B.** CD19+ cell comparisons for the groups with grade 0 and grade I acute urinary adverse effects after completion of radiotherapy; **C.** CD19+ cell comparisons for the groups with grade 0 and grade I acute urinary adverse effects during 23/24 fractions of radiation; [urinary adverse effect (During): acute urinary adverse effects occurring during 23/24 fractions of radiation; urinary adverse effect (Post): urinary adverse effects occurring after completion of radiotherapy.]

**Figure 5:**
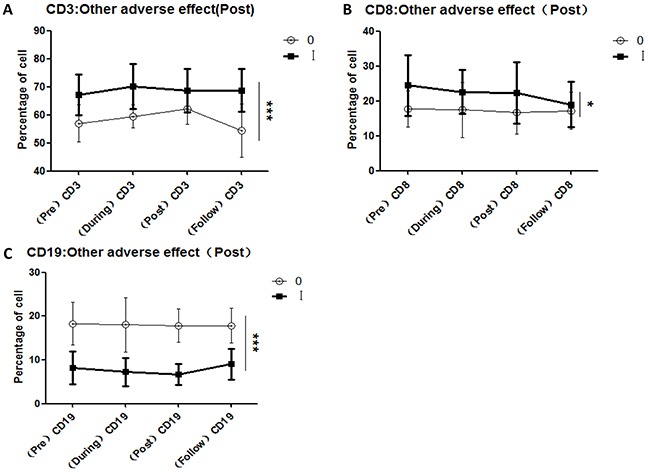
Correlations between variations in lymphocyte subsets and other acute adverse effects induced by CIR **A.** CD3+ cell comparisons for the groups with grade 0 and grade I other adverse effects after completion of radiotherapy; **B.** CD8+ cell comparisons for the groups with grade 0 and grade I other adverse effects after completion of radiotherapy; **C.** CD19+ cell comparisons for the groups with grade 0 and grade I other adverse effects after completion of radiotherapy. [Other adverse effects (Post): other adverse effect occurring after completion of radiotherapy.]

To investigate the reported correlations between lymphocyte subset variations and acute toxicity during radiation (from the first to the final fraction) and post-radiation (from completion of radiation to 3 months later), we compared the variations of all lymphocyte subsets in each patient with the maximum grade of acute hematologic toxicity (Figure 3), acute urinary adverse effects (Figure 4), and acute organ adverse effects (excluding the urinary system) (Figure 5). This comparison was performed for all lymphocyte subpopulations, using separate statistical models. Our results demonstrate that variation in the CD4/CD8 ratio was associated with increased probability of acute hematologic toxicity. In addition, variations in CD3+ and CD19+ cells were closely associated with urinary adverse effects, and variations in CD3+, CD8+, and CD19+ cells were associated with other acute organ adverse effects. Furthermore, the results suggest that the ratios of CD8+ cells during radiotherapy differed (P<0.05) between the groups with grade 0 and grade I-II acute hematologic toxicity, assuming values at pre-radiotherapy of 100. Similar results were obtained for CD8+ cells post-radiotherapy (P<0.01). The group with grade 0 other adverse effects showed a higher ratio of CD3+ cells during radiotherapy than the group with grade I adverse effects (P<0.05), in contrast to observations at follow-up.

### Variations in all lymphocyte subsets correlated with CIR-related parameters

**Figure 6:**
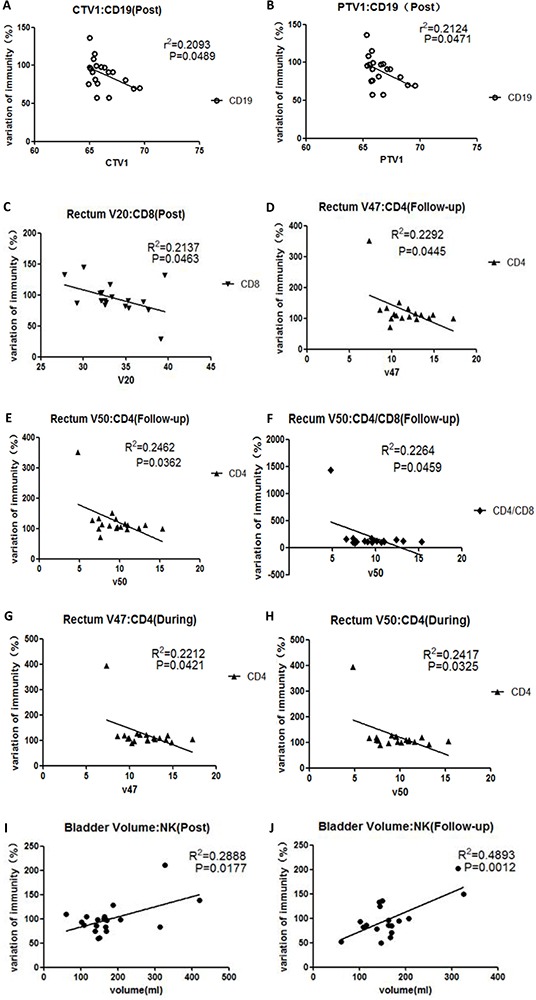
Correlations between CIR-related parameters and variations in lymphocyte subsets at different time points, assuming values at pre-radiotherapy of 100 **A.** presents the relationship between CTV1 and CD19+ cells at post-radiotherapy; **B.** presents the relationship between PTV1 and CD19+ cells at post-radiotherapy; **C.** presents the relationship between Rectum V20 and CD8+ cells at post-radiotherapy; **D**. presents the relationship between rectum V47 and CD4+ cells at follow-up; **E.** presents the relationship between rectum V50 and CD4+ cells at follow-up; **F.** presents the relationship between rectum V50 and CD4/CD8 ratio at follow-up. **G.** presents the relationship between rectum V47 and CD4+ cells during radiotherapy; **H.** presents the relationship between rectum V50 and CD4+ cells during radiotherapy; **I.** presents the relationship between bladder volume and NK cells at post-radiotherapy; **J.** presents the relationship between bladder volume and NK cells at follow-up.

Recently, a number of trials have evaluated the relationship between radiotherapy-related parameters and the influence of radiotherapy on the immune system. In our study, PTV1 and CTV1 correlated with CD19+ cells at post-radiotherapy (Figure 6A, Figure 6B). Rectum V20 correlated with CD8+ cells at post-radiotherapy (Figure 6C); rectum V47 correlated with CD4+ cells during radiotherapy and at follow-up (Figure 6D, Figure 6G); rectum V50 correlated with CD4+ cells during radiotherapy and at follow-up (Figure 6E, Figure 6H); and rectum V50 correlated with the CD4/CD8 ratio at follow-up (Figure 6F). Bladder volume correlated with NK cells at post-radiotherapy and follow-up (Figure 6I, Figure 6J). Other parameters were not correlated with any lymphocyte subset at any time point.

## DISCUSSION

Anti-tumor immunity is an important factor correlated with efficacy of radiotherapy and cancer prognosis. The anti-tumor immune response is involved not only in carcinogenesis, progression, and recurrence of tumors, but also in the treatment and follow-up period. Recent evidence suggests that conventional radiotherapy with photons leads to various degrees of decline in peripheral lymphocytes numbers [29]. Radiotherapy-induced lowered lymphocyte levels have also been observed for prostate cancer [13]. Johnke et al. reported that a reduction in all lymphocyte subsets occurred in stage I to II prostate cancer patients treated with localized radiotherapy [14]. While numerous studies have compared the immune response to conventional photon radiotherapy, few have focused on immunocyte variations following CIR. This study aimed to investigate the changes in lymphocyte subsets after CIR for prostate cancer patients.

Among lymphocytes, NK cells and circulating CD3+, CD4+, CD8+, and CD19+ T lymphocytes are important in anti-tumor immunity. The CD4/CD8 ratio is a sensitive and stable marker of cell-mediated immunity in cancer patients while absolute CD4+ cell counts usually show greater volatility under different physiological conditions. CD19+ cells are recognized as a representative indicator of humoral immunity. Several previous studies suggest that the density and location of infiltrating CD3+, CD8+, and CD45RO+ cells are significant prognostic biomarkers, leading to a new scoring system designated “Immunoscore”, a powerful tool for the classification of malignant tumors [30, 31]. NK and CD8+ T cells also play critical roles in targeting tumors [32].

Our findings show that CD4+ cells and the CD4/CD8 ratio were increased not only during radiotherapy but also throughout the follow-up period, suggesting that CIR-elicited CD4+ T cell activation is persistent. Moreover, the number of CD4+ cells and the CD4/CD8 ratio were slightly higher at post-radiotherapy and follow-up than during radiotherapy. These results may be related to three points. First, carbon ions have a high RBE on the position of the spread-out Bragg peak [33], showing 0.2- to 3.5-fold greater biological effects than equal physical doses of photons. Carbon ions induce tumor death more effectively, which is beneficial to CD4+ T cell activation and proliferation [34]. Second, it is possible that peripheral circulating lymphocytes are directly impacted by the relatively low energy in front of the Bragg peak. Rongjun Liu et al. demonstrated that low-dose total body irradiation greatly increased the CD4+CD44+/CD8+CD44+ effector-memory T-cell number [35]. Third, various parameters of rectum volume correlated with CD4+ variation at different time points (including the follow-up period), CD8+ variation post-radiotherapy, and the CD4/CD8 ratio during follow-up. These results suggest an association with the abundant lymphoid reflux around the rectum. The effect of CIR-induced immunity persisted after treatment completion, which was supported by the correlation of rectum V50 with CD4+ cells and the CD4/CD8 ratio at follow-up. We also found a slight unexplained decrease in CD4+ cells after completion of radiation.

In our study CD19+ cells gradually decreased during radiotherapy (P<0.01), but then rebounded to the pre-radiotherapy levels, suggesting that CIR impacts CD19+ B lymphocytes, which are involved in humoral immunity during radiotherapy. In contrast, CD19+ cells were unchanged after completion of radiation. As we know, the change of lymphocytes is related to radioactive sources, the size of the radiation field, and so on. In the present study, we found that the decrease in CD19+ cells correlated with PTV1 and CTV1, indicating that carbon ions induced the decline in the CD19+ count by direct damage. Thus, humoral immunity is more sensitive to radiation, even with relatively low energy. Furthermore, the CD19+ count correlated with PTV1 and CTV1, but not with the total radiotherapy dose; this could reflect the radiation field, which was equal to PTV1 and CTV1. Limiting the radiation field of the surrounding healthy tissue could help sustain circulating CD19+ lymphocytes.

CD3+ and CD4+ subset counts after 10 fractions of radiotherapy were higher than those before treatment (P=0.005 and P=0.025, respectively) (not shown). Although there was no difference in CD3+ cells among pre-, during-, and post-radiotherapy, the CD3+ cell counts of post-radiotherapy were still significantly higher than those before radiotherapy (P=0.023) (not shown). NK activity, an indicator of immune suppression, decreased and remained depressed for several weeks following photon radiation therapy [11]. However, in our study there was no difference in NK cell counts at any time point after radiation, suggesting that CIR is harmless to innate immunity.

The increased number of CD3+ and CD4+ cells and the CD4/CD8 ratio induced by CIR may be connected with cytokines, which interact with immune cells, enhancing anti-tumor immunity. The two types of T effector cells, CD4+ and CD8+ cells, function as cytokine producers and cytotoxic killer cells, respectively. Th1 cells, one type of CD4+ T cells, secrete Interleukin-2 (IL-2) and interferon-gamma (IFN-β). IL-2 participates in cell-mediated immunity and persistent lymphocytosis could indicate of immune activation by enhancing the synthesis of IL-2 [36]. IFN-β regulates anti-tumor immunity by enhancing the sensitivity of tumor cells to Fas-mediated apoptosis; reducing their ability to evade immune attack; and inhibiting their malignant proliferation [37, 38]. Furthermore, IFN-β can strengthen expression of host T cell receptors and surface MHC antigen expression, as well as tumor necrosis factor concentrations and other anti-tumor responses [39, 40]. Ma et al. measured serum IFN-β in esophageal squamous cell carcinoma (SCC) patients treated with 60-66 Gy radiation and found that IFN-β levels were increased in a dose-dependent fashion [41]. We speculate that these cytokines secreted by T cells play an important role in the changes of lymphocytes counts induced by CIR.

In our study, all prostate cancer patients successfully completed treatment with CIR and were followed for one month. During the follow-up period, the short-term efficacy of radiotherapy was evaluated by PSA serum concentrations, which facilitated the assessment of early stage prostate cancer. Our results demonstrate that the CD4/CD8 ratio and CD19+ cell counts were higher in the PR+CR group than in the SD group, suggesting that stronger immune status predicts the short-term efficacy of prostate cancer patients treated with carbon ions. Shah et al. reported that cervical carcinoma patients with a high CD4/CD8 ratio have better 5-year survival than those with a low CD4/CD8 ratio [42]. In addition, the expression of CD3, CD4 on T cells is known to be a good indicator of overall survival in non-small cell lung cancer patients [27], and increased CD4/CD8 ratio correlates with tumor grade and stage and overall survival [42]. Number of peripheral circulating CD19+ lymphocytes predicts survival in gastric cancer patients [43]. Accordingly, immune suppression may increase the risk of tumor growth, relapse and metastasis [44-46] Thus, we speculated that the number of CD3+, CD4+ cells, CD4/CD8 ratio, and circulating CD19+ lymphocytes could be used to assess prostate cancer progress and prognosis.

Several studies have shown that the immune response is associated with conventional photon radiation-related tissue damage and inflammation. However, the relationship between lymphocyte subset counts and adverse effects after CIR remains unclear. Our results show that higher lymphocyte counts, such as CD19+ cells and the CD4/CD8 ratio, predict lower grade CIR side effects. During and after radiotherapy, increased CD19+ cell counts and CD4/CD8 ratio were associated with minor hematologic toxicity and acute urinary adverse effects, respectively. Additionally, CD19+ cells predict other adverse effects. The possible mechanisms responsible for these findings are worth future exploration.

Our observations have significant implications for prostate cancer patients treated with CIR, who may benefit from an improved cancer immune response. In particular, our analysis of the immune reaction at different time points may help to select patients most likely to benefit from comprehensive treatment and prevent others from suffering unnecessary radiotherapy-related adverse effects. Thus, our present study may pave the way for more effective cancer treatments, such as combined immunotherapy and CIR.

Future work could benefit from a larger number of time points for peripheral lymphocyte subpopulation analysis and enrollment of a larger patient population. Our study demonstrates that CD4+ cells and the CD4/CD8 ratio continued to increase after CIR, even after completion of radiotherapy. However, the temporal pattern of change after each fraction of radiation was not precise. We also observed that CD3+ cell counts were higher after 10 fractions of radiation than the pre-treatment level (P=0.005) (not shown). Conducting peripheral sampling at additional time points on a larger population could help clarify the temporal course of this phenomenon.

## MATERIALS AND METHODS

### Patient selection criteria and treatments

This study included nineteen patients with histopathologically confirmed prostatic adenocarcinoma (cT1–4, any N, any M). Eligible patients were treated with CIR and followed from June to December 2014 at the Shanghai Proton and Heavy Ion Center (SPHIC). Clinical staging was defined according to the AJCC cancer staging criteria (version 1 to 4) and was performed using imaging, including trans-rectal ultrasound (TRUS) and magnetic resonance imaging (MRI), for the detection of tumor size +/− extension. We also performed: bone scintigraphy to detect bone metastases, ^11^C-choline positron emission tomography-computed tomography (^11^C-CHO-PET CT) for local disease staging, CT scans of the chest and abdomen, routine blood tests, blood serum biochemistry profile, prostate-specific antigen (PSA) level, serum creatinine clearance, electrocardiography, complete history assessment, physical examination, and assessment of Karnofsky performance status. Prostate cancer risk staging was based on tumor T stage, initial PSA level, and Gleason score according to the National Comprehensive Cancer Network (NCCN) guidelines.

The treatment plan for each patient was designed by a multidisciplinary tumor board. All participating patients were irradiated with a ^13^C beam with an initial energy of 75 MeV/u (LET = 33.7 keV/μm). A total dose of 63-66 GyE was delivered in 23 or 24 fractions, administered in daily fractions of 2.74GyE (5 times per week) at the established dose point in the planning target volume (PTV).

### Blood sampling and analysis

Peripheral blood samples were obtained pre-radiotherapy, during radiotherapy, post-radiotherapy, and during the follow-up period. The samples were processed immediately for flow cytometric analysis using the BD MultiSET IMK Kit, followed by lymphocyte subset counts of NK, CD3+, CD4+, CD8+, and CD19+ cells using a Cytomics FC500Flow Cytometry System (Beckman Coulter, Inc., Miami, FL, USA) with a 488 nm argon laser.

### Radiotherapy-related toxicity

Acute radiotherapy-related toxicity was assessed after each fraction of radiation and documented according to the Common Terminology Criteria for Adverse Events (CTCAE v4.0). Symptomatic treatment was provided to patients in good status if necessary [37].

### Short-term local efficacy evaluation

Efficacy was evaluated 1, 2, and 3 months after the end of radiotherapy. The efficacy assessment included the following three measures: 1) PSA [47, 48]: A PSA reduction to less than 0.2 ng/ml was defined as a complete response (PSA<0.2 ng/ml, CR); a PSA level lower than the pre-treatment value was defined as a partial response (PR), and a stable PSA level was defined as stable disease (SD); 2) diffusion-weighted imaging apparent diffusion constant (DWI-ADC): In general, regional ADC map values differ depending on location and tissue composition, with malignant lesions showing lower ADC values (approximately 20–40%) than benign or normal prostatic tissue [49]; and 3) ^11^C-CHO-PET: Patients were examined for positive PET findings, and changes were measured according to the standardized uptake value (SUV).

### CIR-related parameters

CIR-related parameters were calculated using the Syngo treatment planning system (Syngo PT Planning, version VB10, Siemens, Germany). Correlations between these parameters and immune variation were analyzed to reveal the possible mechanisms of the immune response to CIR. CIR-related parameters included the PTV; clinical target volume (CTV); bladder_max_ dose, bladder volume, bladder_mean_ dose, bladder V20, V30, V40, V47, V50, V60, and V63; and rectum_max_ dose, rectum volume, rectum_mean_ dose, and rectum V20, V30, V40, V47, V50, and V60. The CTV included the prostate gland with or without the proximal seminal vesicles (CTV1: whole prostate + 2 cm seminal vesicles; CTV2: whole prostate + seminal vesicles, located in front of the anterior wall of the rectum at the same level). The PTV was calculated by adding a 10-mm margin in all directions except posteriorly, where a margin of 5 mm was used.

### Statistical analysis

A Chi-square test was used to compare categorical data. We conducted a Friedman test to analyze variation in lymphocyte subset counts. Linear regression was applied to analyze the correlation between carbon ion beam parameters and variation in lymphocyte subset counts. A Wilcoxon signed-rank test was performed to assess associations between radiation and immune response. Between-group and between-sample differences in numeric data were analyzed with two-tailed Student's *t*-tests. P values less than 0.05 were considered significant for all analyses.

## CONCLUSION

This study demonstrates that variations in peripheral lymphocyte subpopulations have predictive value for the outcome of CIR in prostate cancer patients. However, the complex relationships between lymphocyte variation and radiotherapy-related toxicity, short-term efficacy, and CIR-related parameters require further study.
